# T6. SECOND GENERATION ANTIPSYCHOTIC DRUGS AND MORTALITY: A META-ANALYSIS OF PLACEBO-CONTROLLED RANDOMISED CONTROLLED TRIALS

**DOI:** 10.1093/schbul/sby016.282

**Published:** 2018-04-01

**Authors:** Johannes Schneider-Thoma, Stefan Leucht

**Affiliations:** Technical University of Munich

## Abstract

**Background:**

Despite intensive research it is unknown if treatment with antipsychotic drugs contributes to the reduced life-expectancy observed in patients with schizophrenia and other severe mental disorders. As randomized controlled trials (RCT) are considered the best evidence to examine causal relationship, we aimed to analyze mortality related to antipsychotics based on all placebo-controlled RCTs conducted so far.

**Methods:**

We evaluated mortality in pair-wise meta-analysis of RCTs comparing second-generation-antipsychotics versus placebo across all diagnostic fields.

Information about deaths was extracted from summary data of clinical trials as identified by two searches (last 01/28/2017) in several electronic databases. Furthermore, manufacturing companies and regulatory authorities were contacted, and their websites were searched for trial reports and supplemental information about fatal events. We examined mortality due to any reason (primary outcome), due to natural causes, suicide, and other unnatural causes. We synthesized the results with odds ratios (OR) in a common-effect meta-analysis. We addressed the effects of age, diagnostic field, gender, study duration, antipsychotic drug used, drug dose and polypharmacy with subgroup- and meta-regression-analyses. We used the GRADE framework to evaluate the confidence in the evidence.

**Results:**

We included 596 randomized trials that reported 207 deaths in 53804 patients on placebo (0.38%) and 99 deaths in 31184 patients on drug (0.32%). There was no evidence of significant difference between antipsychotics and placebo regarding mortality due to any reason (OR 1.19; 95% CI 0.93, 1.53), natural causes (OR 1.29; 95% CI 0.85, 1.94), suicide (OR 1.15; 95% CI 0.47, 2.81) and other unnatural causes (OR 1.55; 95% CI 0.66, 3.63). The number-needed-to-harm for all-cause mortality was 6 more deaths in 10 000 drug-treated patients compared to placebo, with a 95% CI ranging from 2 helped to 14 harmed. Most deaths occurred in patients with dementia and these patients also had the highest drug-related mortality in subgroup analysis (OR 1.56; 95% CI 1 .10, 2.21). The OR for patients with schizophrenia (based on 32807 patients) was 0.69 (95 CI 0.35, 1,35).

**Discussion:**

We found no evidence of increased mortality related to treatment with second generation antipsychotic drugs, neither in the overall sample nor in the subgroup of patients with schizophrenia. Results of subgroup analyses indicated however that mortality of patients with dementia might be increased when they are exposed to antipsychotics. Strength of the analysis are the use of data from RCTs and the comprehensive search, which aimed to identify all placebo-controlled RCTs of second generation antipsychotics conducted so far. Limitations of our analysis are the remaining imprecision of the results with a 95% confidence interval including the risk of more than 1 patient harmed in 1000 patients treated with antipsychotics. Moreover, we could only address short-term effects leading to death, but not long-term effects - such as induction of metabolic syndrome – which also contribute to the increased mortality over the whole life-span.

